# “What should I inject next?” Challenging treatment decisions in the multiple anti-VEGF: a review of publications exploring anti-VEGF switching for nAMD

**DOI:** 10.1007/s10792-017-0695-z

**Published:** 2017-08-29

**Authors:** Joseph Pikkel, Shira Attas

**Affiliations:** 1 Ziv Medical Center, Zafet, Israel; 2 Novartis Pharma, Petach Tikva, Israel

**Keywords:** Anti-VEGF, Intravitreal injections, Bevacizumab, Ranibizumab, Aflibercept

## Abstract

**Purpose:**

The purpose of our work was to collate information from studies published to date focusing on switching in anti-VEGF therapy and describe the currently available data on anti-VEGF switching in nAMD.

**Methods:**

A PubMed search of published articles from January 2010 to January 2017 was conducted. Published studies were compared in parameters of sample size, reason for switch, duration of follow-up, and switch outcome (functional and anatomical).

**Results:**

Our search revealed 31 relevant publications. Switching from bevacizumab to ranibizumab mostly resulted in improvement in visual acuity (VA) and anatomical outcomes (CMT, CRT; 7/8 and 6/8 studies, respectively), whereas switching from ranibizumab to bevacizumab was less effective (no VA or anatomical improvement in 2/4 studies). Switching from either agent to aflibercept resulted mostly in improvement of anatomical outcomes (19/21 studies), but rarely in VA improvement (6/21 studies). Not all results were statistically significant, likely due to small sample sizes.

**Conclusion:**

Switching anti-VEGF therapy from bevacizumab to ranibizumab might be of benefit (functionally and anatomically) for patients who failed to improve with intravitreal bevacizumab injections, whereas switching from either agent to aflibercept resulted mostly in reduced macular thickness only.

## Introduction

Age-related macular degeneration (AMD) is the leading cause of irreversible loss of vision in people older than 50 years in the developed world [1]. Neovascular AMD (nAMD) is responsible for almost 90% of severe vision loss in these patients [2, 3].

Anti-VEGF (vascular endothelial growth factor) intravitreal injections are the current standard of care for nAMD: ranibizumab (Lucentis; Genentech, San Francisco, CA) has been established as an effective treatment for nAMD in large-scale, prospective, randomized, controlled multicenter studies. Another anti-VEGF alternative is aflibercept (Eylea; Regeneron Pharmaceuticals, Inc., Tarrytown, NY, USA) which was approved for the treatment of nAMD by the US Food and Drug Administration in 2011. In several countries, the off-label use of bevacizumab (Avastin; Genentech) is the initial anti-VEGF choice due to economic considerations. Several large-scale, prospective, comparative studies demonstrated that bevacizumab is non-inferior to ranibizumab in terms of effect on visual acuity when administered according to the same regimen [4–8].

Bevacizumab and ranibizumab are considered by many physicians as equally safe and effective treatments for nAMD; however, some patients fail to respond even after multiple injections.

The failure of a drug to be effective might be due to either tachyphylaxis, lack of reaction when drugs are used repeatedly over a short period of time, tolerance, or a slow loss of efficacy over time [9]. Tachyphylaxis develops quickly/suddenly and does not improve with dose increase, but the drug’s efficacy might be restored if the drug is stopped and then restarted. On the contrary, with tolerance, improvement might occur with dose increase or shortening the interval between doses, but efficacy is not restored if the drug is stopped and then restarted. Deciding which mechanism is relevant to a specific case can prove to be challenging and oftentimes irrelevant to routine clinical care.

Possible mechanisms for decreased drug response in nAMD might be due to change in the neovascular membrane (more fibrosis), change in lesion type (classic vs. occult), irreversible change in the vessel walls or in neighboring structures (photoreceptors, RPE), or development of chronic inflammatory changes [9–11].

In routine clinical care setting, when confronted with cases of poor initial response or loss of efficacy in a patient who initially responded well to treatment, the retina specialist faces a challenging dilemma. In most cases, clinical decision making is based on personal experience since high-quality publications guiding these treatment decisions are scarce. Some questions which arise in these cases include the following: What benefit would the patient gain from switching to another agent versus continuing care with the current agent? What is the appropriate timing for switching? Which agent to switch to?

Since there are currently no treatment algorithms to guide us through these decisions, we tried to collect data from existing publications describing cohorts of “switch patients” hoping to at least outline what could be the expected outcome of switch from one anti-VEGF agent to another. This work is descriptive in nature and does not attempt to provide recommendations, but rather to trigger a discussion about the clinical usefulness and expected outcomes of switching between anti-VEGF agents in patients with nAMD.

## Methods

A PubMed search of published articles up to January 2017 was conducted. (There were no limitations on past publications.) Key words used were *bevacizumab and ranibizumab* which returned 1318 publications. When the key word *aflibercept* was added, an additional 267 publications were identified. Filtering these 1585 publications by using the key words *anti*-*VEGF switching*, this number was narrowed to 92 (first publications that used these key words appeared in 2012 in PubMed).

**Fig. 1:**
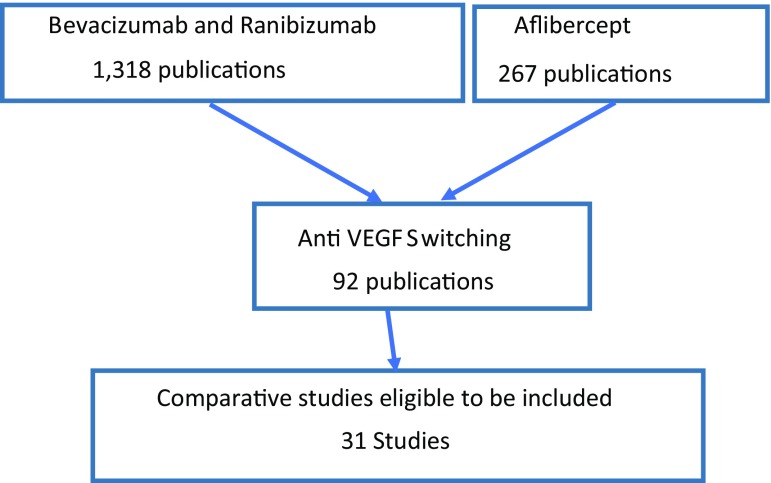
Process of including publications in the analyses

Upon further examination the following publications were excluded: non-peer-review publications, publications published prior to 2010, and case reports and reports where no switching had been reported. Finally, 31 publications were identified as comparative studies eligible to be included in our analysis (Fig. 1).

The following data were collected from these publications:

- Sample size (the number of eyes in the study).
- Reason for switching the anti-VEGF agent.
- Duration of post-switch follow-up.
- Functional outcome (visual acuity improved, no change, or decreased).
- Anatomical outcomes (OCT outcomes improved, no change, or decreased).

The studies were divided into four groups:

- Studies dealing with the switch from bevacizumab to ranibizumab injections (a total of 8 studies).
- Studies dealing with the switch from bevacizumab and/or ranibizumab to aflibercept injections (a total of 16 studies).
- Studies dealing with the switch from ranibizumab to bevacizumab injections (a total of 4 studies).
- Studies dealing with the switch from ranibizumab to aflibercept injections (a total of 6 studies).

## Results

We present our findings in 4 tables according to the four groups of switching. Abbreviations used in all tables are as follows:

VA—visual acuity, CMT—central macular thickness, CFT—central foveal thickness, CME—cystoid macular edema, PED—pigment epithelial detachment, CME—central macular edema, SRF—subretinal fluid, CSF—central subretinal fluid. (We deliberately choose to use the same terms that were used by the authors of the various studies.)

Eight studies reported the outcome of switching from bevacizumab intravitreal injections to ranibizumab intravitreal injections [10, 12–16].

Results of these studies are summarized in Table 1.

**Table 1:** Results of switching from bevacizumab to ranibizumab

Study	Sample size (eyes)	Reason for switch	FU duration	Outcome (mean change)	
Aslankurt et al. [12]	20	Cost and general health insurance applications	21.8 ± 13.1 months	VA	Improved* (−0.2 logMAR)
				CMT	No change (−2 μm)
Ehlken et al. [13]	114	Unresponsiveness to treatment (no improvement or deterioration in VA and morphology)	3 months	VA	Improved* (actual value not reported)
				CFT	Decreased* (−66 μm)
Gasperini et al. [10]	10	Poor response to treatment (lack of definite reduction or an increase in exudation in any compartment)	13 months	VA	Improved (actual value not reported)
				SRF CME PED	Decreased (actual value not reported)
Kaiser et al. [14]	19 (previous treatment: pegaptanib *n* = 1, BCZ *n* = 13, both *n* = 5)	Inadequate clinical response: gain of less than 1 line of VA or a persistence of 300 μm or greater CRT	12 months	VA	Improved (1.17 ± 0.62 ETDRS lines)
				CRT	Decreased (−62.16 μm)
Kent et al. [15]	87	Uniform switch due to pharmacoeconomic governmental decision	39 weeks	VA	Improved* (−0.07 logMAR)
				CRT	Decreased* (−63.6 μm)
Moisseiev et al. [16]	114	Persistent intra- or subretinal fluid and/or the absence of visual improvement	14.2 ± 8.6 months	VA	No change (0.04 logMAR)
				CRT	No change (−28 μm)
Martin Df et al. [17]	57	Persistent intra- or subretinal fluid and/or the absence of visual improvement	12 months	VA	Improved (0.08 logMAR)
Shachat AP [18]	23	Inadequate clinical response: gain of less than 1 line of VA or a persistence of 300 μm or greater CRT	18 months	CRT	Improved (−55 ± µm)
				VA	Improved (0.03 logMAR)
				CRT	Improved (−110 + 56 µm)

* *p* value ≤ 0.05

An additional 4 studies reported the outcome of switching therapy from ranibizumab to bevacizumab intravitreal injections [10, 12, 13, 17].

Results of these studies are summarized in Table 2.

**Table 2:** Results of switching from ranibizumab to bevacizumab

Study	Sample size (eyes)	Reason for switch	FU duration	Outcome (mean change)	
Aslankurt et al. [12]	20	Cost and general health insurance applications	19.7 ± 9.4 months	VA	Improved (−0.03 logMAR)
				CMT	No change (−1 μm)
Ehlken et al. [13]	24	Unresponsiveness to treatment (no improvement or deterioration in VA and morphology)	3 months	VA	No change* (actual value not reported)
				CFT	Decreased (−28 μm)
Gasperini et al. [10]	16	Poor response to treatment (lack of definite reduction or an increase in exudation in any compartment)	13 months	VA	Improved (actual value not reported)
				SRF	Decreased (actual value not reported)
				CME	
				PED	
Pinheiro-Costa et al. [19]	110	Pharmacoeconomic nonmedical board decision	12.2 ± 2.6 months	VA	Decreased* (−2.4 letters)
				CRT	Increased (+19.1 μm)

* *p* value ≤ 0.05

Fifteen articles described switching either from bevacizumab and/or ranibizumab to aflibercept intravitreal injections [20–34]. Previous treatment in this group might have been either bevacizumab or ranibizumab. Some reports included patients for whom treatment was switched to the other drug and then to aflibercept (as third line), while other cases were switched directly to aflibercept. Results of these studies are summarized in Table 3, where previous treatments are documented as well as the final outcome. Changing the treatment strategy was different in terms of timing and cause in the various studies; the reasons for switching and previous agent or agents are detailed in Table 3.

**Table 3:** Results of switching from ranibizumab and/or bevacizumab to aflibercept

Study	Sample size (eyes)	Reason for switch	FU duration	Outcome (mean change)	
Fassnacht et al. [20]	96	Insufficient anatomical response to RBZ/BCZ, defined as any persisting or increasing sub- or intraretinal fluid	16 weeks	VA	Improved (1.9 letters)
				CRT	Decreased* (−39 μm)
Singh et al. [21]	26 (previous treatment: BCZ *n* = 7, RBZ *n* = 17, both = 2)	Responder population to previous anti-VEGF treatment	6 months	VA	Improved* (5.9 letters)
				CRT	Decreased* (−38.6 μm)
Clement et al. [22]	189 (previous treatment: BCZ *n* = 95, RBZ *n* = 84, both = 10)	Non-responders: persistent or recurrent macular edema, SRF, hemorrhage, exudates, and/or PED (82%)	6 months	VA	Improved* (actual value not reported)
		Responders: continued decrease in SRF, cystoid macular edema (CME), macular thickness and/or PED (18%)		SRF	Decreased* (actual value not reported)
				CME	
				PED	
Bakall et al. [23]	36	Recurrent, increase, or persistent subretinal fluid or edema for a minimum of 3 months RBZ/BCZ treatment prior to switching	6 months	VA	No change (0.05 logMAR)
				CMT	Decreased* (−65 μm)
Cho et al. [24]	28	Persistent intra- or subretinal fluid 28–35 days after a minimum of 6 RBZ and/or BCZ injections prior to switching	6 months	VA	No change (0.03 logMAR)
				CSF	Decreased* (−21 μm)
Ferrone et al. [25]	221 (previous treatment: BCZ *n* = 76, RBZ *n* = 145)	Physician’s perception of limited degree or duration of effect, from previous therapy (RBZ/BCZ)	21 weeks	VA	Not change* (actual value not reported)
				CFT	No change* (actual value not reported)
Grewal et al. [26]	21 (previous treatment: BCZ *n* = 4, RBZ *n* = 5, both *n* = 12)	Persistent exudation: intraretinal fluid/cysts, or subretinal fluid (SRF), or both	12 months	VA	No change (−0.02 logMAR)
				CFT	Decreased* (−36.67 μm)
Hall et al. [27]	30 (previous treatment: BCZ *n* = 18, RBZ *n* = 2, both *n* = 10)	Patients who responded well to previous anti-VEGF therapy as well as refractory patients were switched	12 months	VA	No change^†^ (0.015 logMAR)
				CMT	Decreased* (−24 μm)
Messenger et al. [28]	109 (previous treatment: BCZ *n* = 51, RBZ *n* = 40, both *n* = 18)	VA at conversion was ≥20/400	12 months	VA	No change (0 letters)
				CMT	Decreased* (−26 μm)
Oh et al. [29]	96 (previous treatment: BCZ *n* = 30, RBZ *n* = 43, both *n* = 23)	Persistent, recurrent, or worsening exudative fluid or hemorrhage. Patients also were transitioned if they had intolerance to previous anti-VEGF treatments	4 months	VA	No change (0.02 logMAR)
				CFT	Decreased (−18 μm)
Yonekawa et al. [30]	102 (previous treatment: BCZ *n* = 26, RBZ *n* = 48, both *n* = 28)	Refractory: persistent exudation despite monthly injection (*n* = 68)	18 weeks	VA	No change (0.04 logMAR)
		Recurrent: exudation suppressed but requiring frequent injection (*n* = 34)		CMT	Decreased* (−29 μm)
Thorell et al. [31]	73	Persistent or recurrent intraretinal or subretinal macular fluid	6 months	VA	No change (0.5 letters)
				CRT	Decreased* (−19 μm)
Arcinue et al. [32]	63	Multiple recurrences or persistence of exudation following monthly RBZ/BCZ treatments	12 months	VA	No change (−2 letters)
				Maximum retinal thickness	Decreased* (−107 μm)
Homer et al. [33]	21	Patients who required treatment on a 4–8-week interval to remain exudation-free (on an OCT-guided T&E protocol)	24 months	VA	No change (0.0 logMAR)
				CST	No change (−8.5 μm)
Pinherio-Costa et al. [34]	85	Refractory and recurrent AMD	14.7 months	VA	Decreased (−2.1 letters)
				CRT	Decreased* (−79.2 μm)
Pfau et al. [35]	96	Injection interval of less than 6 weeks or permanently persisting intra- and/or subretinal fluid or persistent pigment epithelial detachments	12 months	VA	No change
				CRT	Decreased (− 31.36 µm; SD ± 70.64 µm)

*BCZ* previous treatment with bevacizumab, *RBZ* previous treatment with ranibizumab

* *p* value ≤ 0.05

^†^BCVA improved significantly at 6 months (from 20/64 to 20/52, *p* = 0.036), but showed no improvement at 12 months of follow-up (from 20/64 to 20/66)

Six studies were identified which described switching a homogenous cohort from ranibizumab as first-line treatment to aflibercept as second-line treatment (Table 4) [33–38].

**Table 4:** Results of switching from ranibizumab to aflibercept

Study	Sample size (eyes)	Reason for switch	FU duration	Outcome (mean change)	
Gharbiya et al. [36]	31	Persistent intraretinal or subretinal fluid with or without PED following RBZ treatments	6 months	VA	No change (0.3 letters)
				CSF	Decreased* (−180 μm)
Heussen et al. [37]	12	Diminishing effect over time or persistent intra- or subretinal fluid following RBZ treatments	4 injections after the switch	VA	Improved (−0.22 logMAR)
				CSF	Decreased* (−67 μm)
Kumar et al. [38]	34	Persistent subretinal and/or intraretinal fluid despite previous RBZ treatments	6 months	VA	Improved* (−0.1 logMAR)
				CFT	Decreased* (−168 μm)
Kawashima et al. [39]	15	Recurrent or residual exudative changes after the last three RBZ injections	6 months	VA	No change (0.01 logMAR)
				CRT	Decreased* (−71 μm)
Batioglu et al. [40]	29	Persistent intraretinal or subretinal fluid and PED following RBZ treatments	4.55 ± 2.14 months	VA	No change (−0.06 logMAR)
				CMT	Decreased* (−126 μm)
Gerding et al. [41]	40	Persistent or recurrent intra- and/or subretinal fluid	6 months	VA	Improved* (0.65 ETDRS lines)
				CFT	Decreased* (−96 μm)

* *p* value ≤ 0.05

Overall, switching from bevacizumab to ranibizumab resulted in VA and anatomical improvement in the majority of studies (7/8 and 6/8 studies, respectively), whereas switching from ranibizumab to bevacizumab was less effective (no VA or anatomical improvement in 2/4 studies).

Switching from either agent (bevacizumab and/or ranibizumab) to aflibercept resulted in improvement of retina anatomy in most cases (20/22 studies), but rarely in VA improvement (6/22 studies).

## Discussion

Despite the well-proven efficacy of anti-VEGF agents in treating nAMD, not all patients experience the desired extent of functional and anatomical improvement. This could prove to be a very frustrating situation for both treating retinal specialist and patient, as the treatment and follow-up can be cumbersome, and failing to achieve the desired result may lead to loss of confidence and reduced compliance to the follow-up and treatment regimen.

With several available anti-VEGFs on the market, patients with unsatisfactory responses to one anti-VEGF can readily be switched to another.

Our literature review focused on analyzing the response of switching resistant nAMD patients from the initially chosen anti-VEGF (by the judgment of the treating retina specialist) to another anti-VEGF agent, but we also concluded results of switching anti-VEGF agents due to other reasons such as economic considerations or regulatory/insurance decisions. It is important to note that this is not a head-to-head comparison and caution should be taken when comparing results from different studies. Another important disclaimer is the fact that all of these studies are retrospective in nature, with an inherent patient selection bias.

Our analyses revealed 31 relevant publications. In most studies, switching from bevacizumab to ranibizumab showed improvement in VA and reduction in anatomical features (7/8 and 6/8 studies, respectively), whereas switching from ranibizumab to bevacizumab was less effective (no VA or anatomical improvement in 2/4 studies). Switching from either agent to aflibercept generally resulted in anatomical improvement (19/21 studies), but rarely in functional improvement (6/22 studies). To date, there are no large data available on direct switch from bevacizumab to aflibercept.

Similarly, a meta-analysis of seven retrospective and prospective studies indicated that following treatment switch from ranibizumab or bevacizumab to aflibercept, resistant nAMD patients may have a significant improvement in CRT, while the VA was mostly stabilized after 6-month follow-up [42].

Since the desired outcome would be of sustained change rather than a temporary improvement, the available duration of post-switch follow-up is a key consideration when analyzing results.

This was illustrated by Hall and colleagues who reported significant improvement in BCVA at 6 months (from 20/64 to 20/52, *p* = 0.036), but no such improvement was recorded at 12 months of follow-up (from 20/64 to 20/66) [27].

There are now emerging data on “switchback” cohorts, demonstrating that while switching may have an effect it may be limited in nature, and there are situations where switching back to an agent which was deemed ineffective in the past may actually produce clinically significant results. This was recently reported by Despreaux and colleagues in a study where 47 eyes with nAMD were switched back from aflibercept to ranibizumab demonstrating a short-term benefit of this switchback in patients who had shown no benefit from the initial switch from ranibizumab to aflibercept [43].

One major limitation to these studies is the lack of uniform guidelines for switching treatment and also the pooling of data from heterogeneous patient cohorts that were treated by different retina specialists. In most cases, these publications provide little or no information regarding the treatment regimen prior to or after the switch. This of course may impact both functional and anatomical outcomes and be significant in the definition of treatment failure. Also, the timing of switch is not provided or is not uniform, making it difficult to draw conclusions on the optimal time for treatment switch, which could potentially be after 3 injections, 6 injections, or perhaps more. All of these are crucial questions in the current clinical environment, and additional well-designed larger studies would be needed to answer them.

In conclusion, switching anti-VEGF agents from bevacizumab to ranibizumab may be of benefit for patients who fail to improve with intravitreal bevacizumab injections. Ranibizumab was shown in various the publications included in this analysis as a good alternative treatment in nAMD after bevacizumab failure. When switching from either bevacizumab or ranibizumab to aflibercept, anatomical improvement was seen in most cases, but only a minority of publications described improvement in functional outcomes. To date, there are no data available on direct switch from bevacizumab to aflibercept.

While we do not aim to provide a definitive treatment guideline as to “what to inject next,” this publication review may be useful to manage our expectations as retina specialists, as well as inform our patients of what to expect when treatment switch is recommended.
